# Treatment of vulvar basal cell carcinoma with Slow‐Mohs micrographic surgery A case report

**DOI:** 10.1002/ccr3.6442

**Published:** 2022-10-13

**Authors:** Ali Asilian, Reza Moeine, Hossein Hafezi, Reza Shahriarirad

**Affiliations:** ^1^ Department of Dermatology, Skin Diseases and Leishmaniasis Research Center Isfahan University of Medical Sciences Isfahan Iran; ^2^ Thoracic and Vascular Surgery Research Center Shiraz University of Medical Sciences Shiraz Iran

**Keywords:** basal cell carcinoma, mohs micrographic surgery, treatment, vulva

## Abstract

Vulvar Basal Cell Carcinoma (BCC) accounts for only 0.4% of all BCCs. We present a case of BCC that developed on the vulvar area with a pinkish lesion and pruritus for about 2 years and was successfully treated with Mohs micrographic surgery.

## Graphical abstract

Regarding the usual delayed diagnosis of the vulvar basal cell carcinoma along with the importance of tissue preservation in this sensitive area, Mohs micrographic surgery is the treatment of the choice for the vulvar basal cell carcinoma.

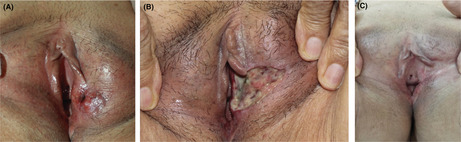

## 1 INTRODUCTION

Basal cell carcinoma (BCC) is the most common skin cancer. It mainly occurs in sun‐exposed areas with nearly 85% of the BCCs located on the head and neck region.^1^, ^2^, ^3^

Vulvar BCC is rare and accounts for less than 0.4% of all BCCs^1^; only 2–5% of all vulvar malignancies are BCC.^2^ Affected women are typically white, with a mean age of 70.35 years.^4^ Given the fact that BCC appearance typically correlates with the cumulative ultraviolet radiation, its development on sun‐protected areas like the vulva suggests other etiologies.^4^ Immunosuppression, chronic irritation, pelvic radiation, and trauma might be contributing factors.^5^ In this report, we present a case of BCC that developed on the vulvar skin and discuss its management.

## 2 CASE PRESENTATION

**FIGURE 1:**
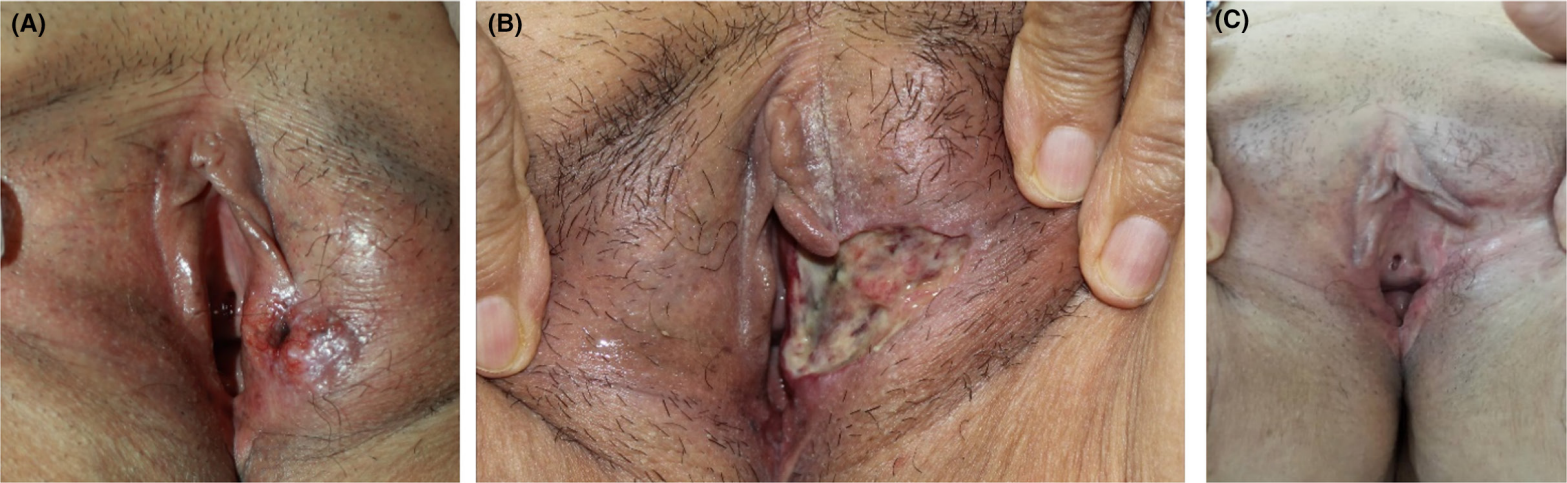
Erosive plaque on the right labia major in a 77‐year‐old female in favor of basal cell carcinoma (A) On admission, (B) After Mohs surgery, and (C) After 13 months of follow‐up

A 77‐year‐old, multiparous woman presented with vulvar discomfort and itching. She had a pink lesion that had started 2 years earlier and progressed insidiously (Figure 1). She tried corticosteroid and anti‐fungal creams as a treatment without any success. The patient’s history was negative for routine medication use, drug abuse, sexually transmitted diseases, tobacco smoking, immunosuppression, or any specific disorder. She also denied any personal or family history of skin cancers or internal malignancies.

In physical examination, a 10 × 4 cm, well‐demarcated, erosive plaque was observed on the right labium majus. There was no palpable lymph node in the inguinal and femoral regions. No suspicious lesions were found on complete skin examination. Ultrasound also showed no abnormal inguinal lymph nodes.

**FIGURE 2:**
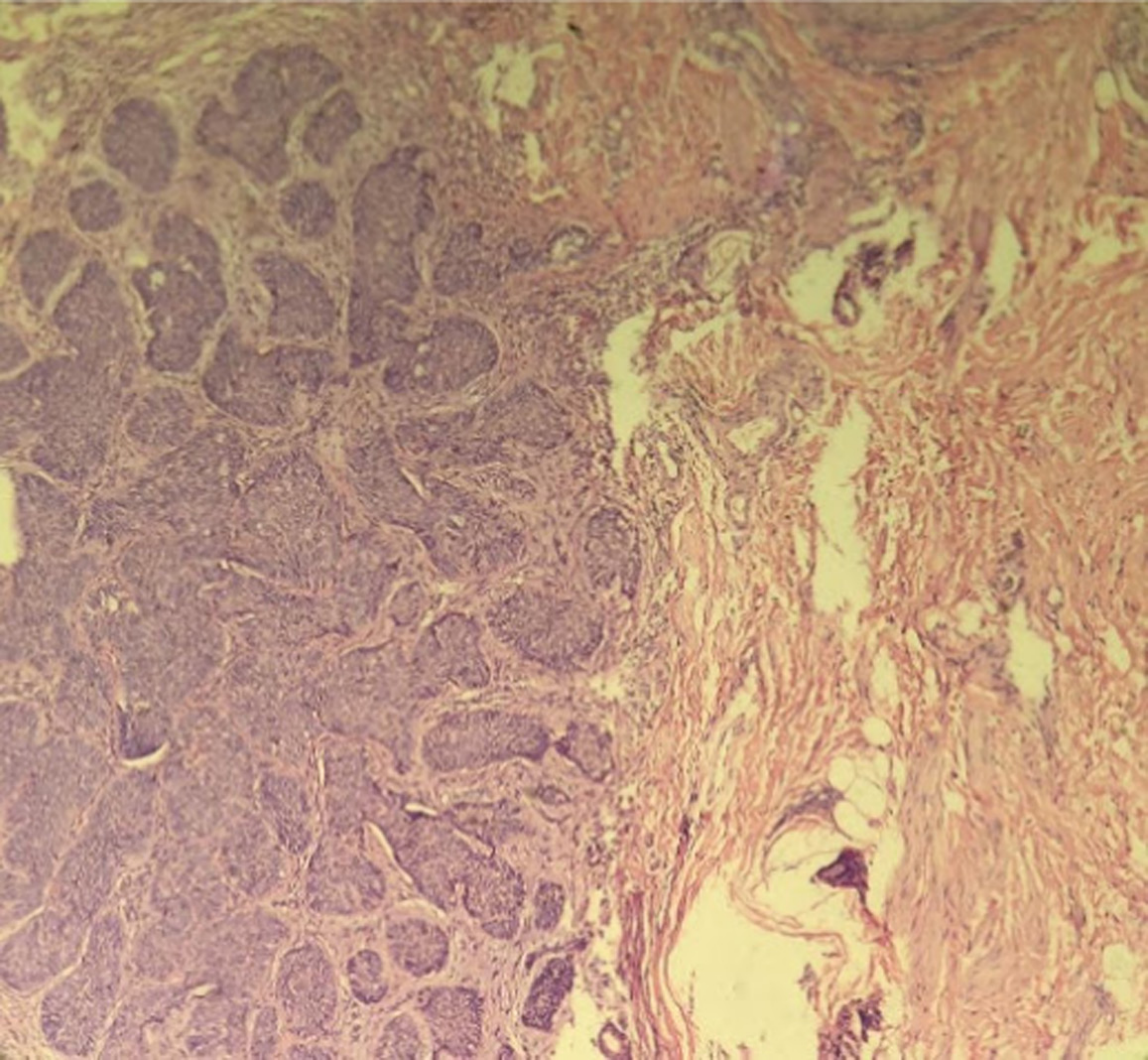
A pathological section demonstrating the dermal neoplastic proliferation of small epithelial cells with a high‐nuclear‐to‐cytoplasmic ratio as irregular nests and palisading of the cells at the periphery, in favor of the basal cell carcinoma

To confirm the diagnosis, an incisional biopsy of the lesion was performed. Pathology of the lesion showed dermal neoplastic proliferation of small epithelial cells with a high ‐nuclear‐to‐cytoplasmic ratio as irregular nests and palisading of the cells at the periphery (Figure 2). The diagnosis of nodular BCC was confirmed and the patient underwent Slow Mohs micrographic surgery (MMS). At 13 months of follow‐up, the area was completely healed and there was no sign of recurrence. (Figure 1).

## 3 DISCUSSION AND CONCLUSION

Due to its rare occurrence, BCC of the vulva is poorly studied and reported. Etiologic factors include those shared among cutaneous BCC, i.e., radiation therapy, exposure to coal tar or arsenic, burns, traumatic scars, chronic skin irritation, certain types of immunosuppression, hereditary skin conditions such as xeroderma pigmentosum and nevoid basal cell carcinoma syndrome; some cases have been associated with the Paget disease of the vulva and lichen sclerosis.^1^ However, in our case, we did not find any relevant risk factors for the development of BCC.

Inconsistent with our case report, a review of the literature showed a delay of several years between the development of the vulvar BCC and the appearance of its symptoms such as burning and itching.^3^

The clinical manifestations of vulvar BCC are non‐specific and include itching, discomfort, bleeding, pain, infiltrating‐pigmented lesions, and the presence of a palpable mass in the vulvar region.^6^ In most women, they are located on the non‐mucosal surface of the labia majora. Bilateral or multifocal vulvar BCCs are rare.^3^

In general, the occurrence of metastasis is rare. Aggressive histological patterns (morphea form, infiltrating, and basosquamous), the large size of the primary tumor (>2 cm), and occasional perineural extension seem to be a common denominator in cases of metastatic vulvar BCC^7^, ^8^ with the most common sites of metastasis include inguinal nodes, the bone, lung, and skin.^7^

Biopsy of suspicious lesions is the best diagnostic method.^5^ Differential diagnosis of vulvar BCC includes inflammatory dermatoses such as atopic dermatitis, psoriasis, lichen planus, lichen simplex chronicus, other vulvar malignancy such as vaginal intraepithelial neoplasia, vulvar Paget's disease, squamous cell carcinoma and benign tumors of the vulva (such as flat seborrheic keratosis).^7^

Treatment of BCC can be surgical or non‐surgical. Surgical techniques include curettage, cautery, cryosurgery, MMS, and complete surgical excision; nonsurgical options include methods such as radiation therapy and systemic chemotherapy.

The most reliable treatment for basal cell carcinoma is surgery. Methods such as curettage, cautery, and cryosurgery have been used to treat superficial lesions; however, patients’ compliance with these procedures could be reasonably poor. In addition, BCC lesions are moderately sensitive to radiation therapy and systemic chemotherapy is not useful for the treatment of localized BCC.

Wide surgical excision, with the aim of clear histological margins, is considered an adequate treatment for most instances of vulvar BCC. To prevent local recurrence, the surgery should be designed to remove 5–10 mm of the normal‐appearing periphery and depth down to the fascia.

Due to incomplete excision, local recurrence of vulvar BCC has been reported to be as high as 10%–20%; therefore, MMS could be considered as a promising treatment for vulvar BCC.

The MMS has been particularly used for the treatment of the recurrent BCC, large BCC, or histologically aggressive BCC, providing cure rates of more than 97% for these cases. This procedure is associated with better cosmetic and functional outcomes.^6^, ^7^, ^9^, ^10^

In the current case, we used MMS for the treatment of the vulvar BCC with good cosmetic results. MMS also provides good margin control for prevention of recurrence and therefore can be regarded as a procedure of choice, especially regarding usual delay presentation, for treatment of the vulvar BCC.

We utilized a delay Mohs (Slow Mohs) method, in which initially the tumor is debulked, and then the 2 mm of the borders are resected with a 45‐degree angle, and subsequently, the tumor is divided into two or more sections (based on the size of the tumor) and numbered accordingly clockwise. Each section border is then colored and placed in formalin and sent for pathological evaluation through a horizontal slice, to determine tumoral involvement. The difference of this technique with conventional Mohs, which is through a frozen section evaluation. Our technique has demonstrated better results regarding the pathological results, which therefore provides better treatment and follow‐up results compared to the conventional MMS technique with frozen section.^11^

## AUTHOR CONTRIBUTIONS

A.S. and R.M. diagnosed the case and carried out the treatment. F.I. and H.H. were major contributors in the case management and data collection. R.S drafted the manuscript. All the authors read and approved the final manuscript.

## FUNDING INFORMATION

No financial support was received for this case report.

## CONFLICT OF INTEREST

The authors declare that they have no competing interests.

## ETHICAL APPROVAL

Written informed consent was obtained from the patients in our study. The purpose of this research was completely explained to the patient and they were assured that their information will be kept confidential by the researcher. The present study was approved by the Medical Ethics Committee of the academy.

## CONSENT

The written informed consent was obtained from the patient for publication of this case report and any accompanying images. A copy of the written consent is available for review by the Editor of this journal.

## Data Availability

All the data regarding this study has been reported in the manuscript. Please contact the corresponding author if you are interested in any further information.
